# Cell-Mediated Immunity and Vaccines

**DOI:** 10.1155/2014/632632

**Published:** 2014-03-05

**Authors:** Jaya Kumari, Senthamil R. Selvan, Stephane Becart, Subhasis Chattopadhyay, Roy Ambli Dalmo

**Affiliations:** ^1^Norwegian College of Fishery Science, Faculty of Biosciences, Fisheries and Economics, University of Tromso, 9037 Tromso, Norway; ^2^Nofima, Muninbakken 9-13, 9291 Tromso, Norway; ^3^Division of Solid Tumor, Department of Medical Oncology, Thomas Jefferson University, Philadelphia, PA 19107, USA; ^4^Janssen Research & Development, LLC, Immunology Discovery, 3210 Merryfield Row, San Diego, CA 92121, USA; ^5^School of Biological Sciences, National Institute of Science Education and Research (NISER), Bhubaneswar, Odisha 751005, India

Cell-mediated immunity (CMI) is one of the important effector arms during immunological responses to infection and vaccine development. Therefore, a key challenge in developing new vaccines that are effective involves the induction of optimal memory T cell responses [1]. This requires a better understanding of the mechanisms and signals involved in the generation and maintenance of the host CMI response. Thus, a rational strategy should be designed based on crucial factors such as the level and duration of antigen exposure, the use of adjuvants that can enhance CMI and antibody response, and also establishing accurate correlates of protection from diseases. The papers presented in this special issue focus on the leveraging knowledge of CMI and its probable role in novel vaccine strategies.

C. E. Jäkel et al. reviewed twenty recent clinical studies regarding the application of cytokine-induced killer (CIK) cells for the treatment of gastric, pancreatic, hepatocellular, and colorectal cancers. They showed that in all studies, CIK cell therapy was well tolerated and safe as well as superior to conventional therapies alone, since CIK cells have a favorable therapeutic effect with non-MHC-restricted tumor targeting and uncomplicated isolation and cultivation. The clinical study reviewed here thus provides a promising approach in cancer therapy.

Since the last decades of cancer research, numerous approaches have been initiated aiming at activating cytotoxic immune reactions against tumors. Besides targeting the adaptive immune system, stimulators of the innate immune system gained much attention. In this context and resulting from their strong immune stimulatory capacity, ligands for Toll-like receptors (TLRs) were extensively studied. In line with this, two of the research papers deal with those aspects of TLR ligands that have hitherto not been explored in great depth. The paper by Naumann et al. explores the physiochemical and immunostimulatory properties of RGC100, a TLR3 agonist, which may thus represent a promising adjuvant for prophylactic and therapeutic vaccination strategies. They showed that RGC100 has a defined chemical structure and length and has good solubility and stability as well as induces CD1c+ DC driven T cell proliferation. Furthermore, S. Stier et al. clarified the two-sided roles of TLRs expressed by tumor cells. Utilizing the potential of various TLR ligands, alone or in combinations, they demonstrated control of tumor growth and activation of immune cells but also unraveled the requirement of choosing the right combinations as certain combinations accelerated the tumor growth. The findings underscore the rationale for using TLR ligands in cancer immunotherapy, preferably together with conventional chemotherapy, as strongest oncolytic effects were observed in the presence of a functional immune system. Further, their studies offer insights for elucidating the exact balance between pro- and antitumor activities of TLR agonists as single agents but especially of combinations.

S. L. Yingst et al. raise new questions about the importance of CD8+ T cells in defense against *Brucella* infection. They showed a limited role of CD8+ T cells, as IFN-*γ* producers or cytotoxic cells, in secondary immunity to *B. melitensis *in contrast to previous studies, focused on *B. abortus*-induced CD8+ T cell responses. Therefore, the authors suggest that a vaccine strategy aimed at sensitizing CD8+ T cells may have limited value, although future investigation is necessary.

New insights on the interaction between *Haemophilus parasuis* and cytokine expression in pigs for better understanding Glasser's disease pathogenesis and effective vaccine development are described in the paper by R. Frandoloso et al. In this context, they describe the capability of subunit vaccines (NPAPTim and NPAPTit: native proteins with affinity to porcine transferrin) to dampen the transcription of several chemokines and cytokines (CCL-2, CXCL-8, IL-1*α*, IL-1*β*, IL-6, IL-10, and TNF-*α*) directly related to severe inflammation in systemic and target infection organs of nonimmunized animals. This highlights that NPAPT antigens might be a suitable candidate to control Glasser's disease caused by *H. parasuis* Nagasaki strain.

We hope the collection of papers in this special issue will enrich our readers and researchers with latest information with respect to wide but important field of cell-mediated immunity and its relationship with novel vaccine development.
